# Early Life Domestic Pet Ownership, and the Risk of Pet Sensitization and Atopic Dermatitis in Preschool Children: A Prospective Birth Cohort in Shanghai

**DOI:** 10.3389/fped.2020.00192

**Published:** 2020-04-24

**Authors:** Chunxiao Li, Qian Chen, Xi Zhang, Huaguo Li, Quanhua Liu, Ping Fei, Lisu Huang, Zhirong Yao

**Affiliations:** ^1^Department of Dermatology, Xinhua Hospital, Shanghai Jiaotong University School of Medicine, Shanghai, China; ^2^Institute of Dermatology, Shanghai Jiao Tong University School of Medicine, Shanghai, China; ^3^MOE-Shanghai Key Laboratory of Children's Environmental Health, Xinhua Hospital, Shanghai Jiao Tong University School of Medicine, Shanghai, China; ^4^Clinical Research Center, Xinhua Hospital, Shanghai Jiao Tong University School of Medicine, Shanghai, China; ^5^Department of Pediatrics, Xinhua Hospital, Shanghai Jiao Tong University School of Medicine, Shanghai, China; ^6^Department of Ophthalmology, Xinhua Hospital, Shanghai Jiao Tong University School of Medicine, Shanghai, China; ^7^Department of Pediatrics Infectious Diseases, Xinhua Hospital, Shanghai Jiao Tong University School of Medicine, Shanghai, China

**Keywords:** pet ownership, dog ownership, atopic dermatitis, allergic sensitization, birth cohort

## Abstract

**Background:** Although domestic pet ownership is on the rise, the impact of early life pet ownership on children's pet sensitization and atopic dermatitis (AD) remains controversial.

**Methods:** Shanghai Allergy Cohort is an ongoing prospective study followed up to the age of 5 years. Pregnant mothers were recruited and their offspring were followed up every year by a group of pediatricians. Information on furred pet ownership was collected by the questionnaire. AD was diagnosed by dermatologists according to disease history and Williams criteria at 5 years ± 1 months. Skin prick test (SPT) was performed to determine sensitization to specific allergens. Multiple logistic regression models were used to evaluate the associations between pet ownership and AD, dog/cat sensitization.

**Results:** In the 538 children at preschool age, 112 (20.82%) were diagnosed with AD. Dermatophagoides pteronyssinus and Dermatophagoides farina were the most common allergens, and almost 10% of children were positive to dog and cat. The percentage of positive SPT reactors at 5-year old was 65.28% in the group of children with AD, higher than that in non-AD group (44.57%). Domestic pet ownership at both infant and preschool period was positively associated with an increased risk of sensitization to dog (OR _adjusted_ = 2.85 [95% CI: 1.08–7.50 for infant exposure], OR _adjusted_ = 2.73 [95% CI: 1.33–5.61] for preschool exposure), and interestingly, pet ownership at infant period negatively associated with higher risk of AD at 5-year old (OR adjusted = 0.33 [95% CI: 0.12–0.88]).

**Conclusion:** This is the first prospective birth cohort study in Shanghai that found half of preschool children had positive allergen sensitization even in the non-AD children. Although early life exposure to dog may increase the risk of dog sensitization, it significantly decreased the risk of AD. The underlying mechanisms warrant further investigations.

## Introduction

In the recent decades, the prevalence of atopic dermatitis (AD) in China rapidly increased from 3.07% in 2002 to 8.30% in 2012 and even arrived at 12.94% in 2015 (1–3). Meanwhile, raising a pet especially a dog, becomes more popular among Chinese families in metropolitans like Shanghai. It is reported that domestic animals have become a major source of indoor allergens in urban areas of China (4). However, the prevalence of pet allergen sensitization is limited especially among healthy pre-school children. It's also not clear whether raising pet will increase the risk of allergen sensitization.

Epidemiological evidence of the relationship between pet exposure and risk of AD among children from prospective studies was controversial (5–9). Earlier reviews as well as cohort studies showed a protective effect of pet exposure on the risk of AD in infants or children (9–11). However, other studies found pet-keeping had no or increased effect on allergic disease (12, 13). Ambiguous results have also been shown in previous studies of pet exposure on the risk of pet sensitization, with decreased risk (14, 15), increased risk (16, 17), and no effect (16–18) of such exposure. The inconsistencies or discrepancies among these studies could be due to the differences in the definitions of pet exposure, methods and age of AD diagnosis in children.

In this birth cohort study, with thorough physical examination and lab tests like skin prick test, we aimed to find the impact of furred pet exposure in early life on the risks of AD and pet sensitization.

## Methods

### Study Design

The study was conducted according to the guidelines laid down in the Declaration of Helsinki, and all procedures were approved by the institutional review board of Xinhua hospital and the International Peace Maternity and Child Hospital.

**Figure 1:**
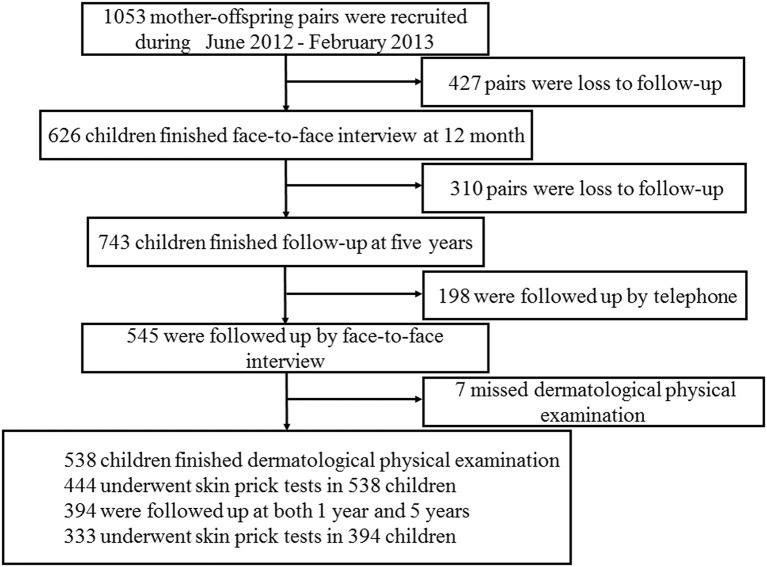
Flow chart of the follow up.

Shanghai Allergy Cohort is an ongoing birth cohort study with the pursuit of early life risk factors of allergic risks. A total of 1,053 mother-newborn pairs were enrolled in our study as a baseline from Xinhua Hospital and the International Peace Maternity and Child Hospital affiliated to Shanghai Jiao Tong University between June 2012 and February 2013 in Shanghai, China. The detailed recruitment strategies and characteristics of the cohort have been described previously (19). Briefly, mothers were recruited during pregnancy and children were followed up each year face-to-face or by telephone. At each visit, a standard questionnaire interview was conducted to collect children's information regarding home environmental factors. Of these pairs, 626 children finished face-to-face interviews at 12 months and 743 pairs (70.56%) completed the 5-year follow-up (including 198 by telephone interview). The physical examination was performed in 545 children by a group of physicians including pediatrician, dermatologist, respiratory physician, gastroenterologist, and ophthalmologist. A full skin examination, including recording of all positive signs of skin and rash scoring in different parts of the body, was completed in 538 children who were included in the analysis. The follow up flow chart is shown in Figure 1.

Information on maternal educational level, maternal history of allergic disease, prenatal furred pet exposure, maternal passive tobacco smoke exposure during pregnancy, mode of delivery, birthweight, parity, breastfeeding, siblings, and gestational age at birth was collected using self-administered questionnaires. Raising any furred pet, mainly dog, cat and rabbit, indoor for at least 1 month was defined as raising a furred pet. AD was diagnosed according to disease history and the criteria of Williams published in 1994 (20). SPT was performed by dermatologists to test specific allergens, including nine aeroallergens [*Dermatophagoides pteronyssinus(der p), Dermatophagoides farina(der f)*, dog dander, cat dander, *Aspergillus niger*, ragweed pollen, *Platanus hispanica* pollen, birch pollen, and willow pollen] and ten food allergens (egg, milk, mango, prawn, sea crab, beef, mutton, cashew, walnut). Histamine and physiologic saline were used as positive and negative controls, respectively. A positive result was confirmed when diameters of the urticarial weal was at least 3 mm larger than that of negative control. A diagnosis of AD required the presence of the symptom of an itchy rash as well as at least 3 of the following features: (1) history of flexural involvement; (2) onset under the age of 2 years; (3) personal history of asthma or allergic rhinitis; (4) history of a dry skin; and (5) visible flexural dermatitis. The SCORAD index was used to evaluate the severity of AD as previously described by Schmitt (21).

### Statistical Analysis

Categorical variables were described using frequencies and percentages. The *chi-*square test was used to evaluate the association between dog sensitization and AD. Non-parametric Mann-Whitney-Wilcoxon test was applied to compare the difference in SCORAD between AD patients with furred pet exposure and those without (21). Associations between pet exposure in pregnancy, infancy, and the preschool period and risk of AD at the age of 5 years were evaluated by multivariate logistic regression models adjusted for sex, maternal atopy, maternal education, mode of delivery, birthweight, parity, gestational age, breastfeeding, siblings and maternal tobacco smoke exposure during pregnancy. The impact of raising pet on allergy sensitizations were also evaluated. All statistical analyses were performed with SPSS (version 20, Chicago, IL, USA).

## Results

### Demographic Characteristics

A total of 743 (70.56%) children finished the follow-up at 5 years of age; among them, 198 children were followed-up by telephone and 545 by face-to-face interview. We finally included 538 children who finished both the questionnaire and physical examination, 97.03% of these children were born at full-term and 92.57% were first-parity birth (Table 1); 71.00% were breastfed to at least 6 months. A total of 11.16% (60/538) owned pets at home during pregnancy, mainly dogs (88.39%). During the first year after birth, 13.96% (55/394) of children had raised pets indoor for at least 1 year, mainly dogs (82.14%, 45/55). During the preschool period, 17.10% of children owned pets at home, and still mainly dogs (55.55%). The number of families with cat exposure was small (0.93%, 5/538) but among them only one child was diagnosed with AD. No differences were found in the characteristics between pet ownership group and non-pet ownership group (Table 1).

**Table 1:** Demographic characteristics of mother-child pairs in Shanghai Allergy Cohort by pet owner.

**Characteristics**	**N (%)**	**Non-Pet owner**	**Pet owner**	***p***
**N**	538	447	92	
**Boys**	272 (50.56)	224 (50.11)	42 (45.65)	0.41
**Maternal educational level**				0.15
Lower than bachelor	75 (13.94)	61 (13.68)	13 (14.13)	
Bachelor	411 (76.39)	336 (75.33)	75 (81.52)	
Higher than bachelor	53 (9.85)	49 (10.99)	4 (4.35)	
**Mode of delivery**				0.46
Vaginal delivery	139 (25.84)	118 (26.58)	21 (22.82)	
Cesarean section	397 (74.1)	326 (73.42)	71 (77.17)	
**Birth Weight**				0.53
<2,500 g	14 (2.61)	12 (2.70)	2 (2.17)	
2,500–4,000 g	472 (87.90)	388 (87.19)	84 (91.3)	
>=4,000 g	51 (9.50)	45 (10.11)	6 (6.52)	
Primiparity	498 (92.57)	413 (92.8)	85(92.4)	0.89
**Gestational age**				0.87
≥ 37 weeks	522 (97.03)	432 (96.86)	90 (97.83)	
<37 weeks	16 (2.97)	14 (3.14)	2 (2.17)	
Passive smoking during pregnancy	164 (30.48)	130 (29.08)	34 (36.96)	0.34
Maternal atopy	87 (16.17)	73 (16.33)	15 (16.30)	0.97
Breastfeeding before 6 months	382 (71.00)	319 (71.36)	63 (68.48)	1.00

### Prevalence of Positive Allergic Sensitization and Associations Between Early Life Pet Ownership and Allergies Sensitization at Age 5 Years

**Figure 2:**
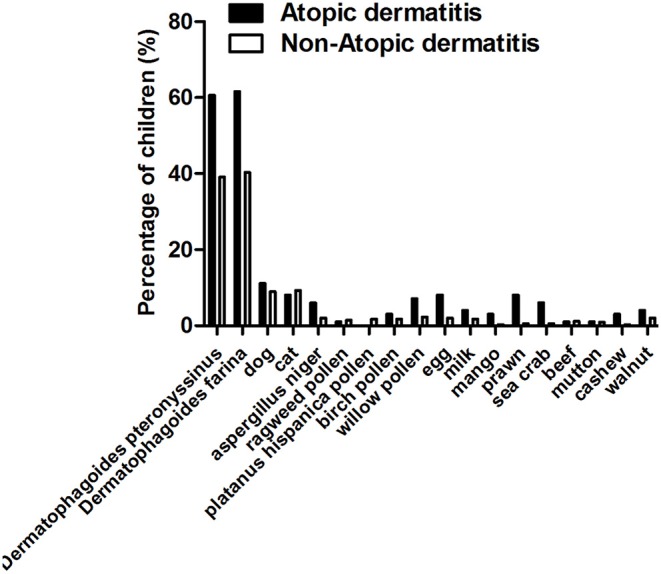
Frequency of positive allergic sensitization in Shanghai Allergy Cohort stratified by AD. The particular type of allergy sensitization in each group was calculated. Percentages of children with positive allergic sensitization in either group were analyzed. The x-axis shows the particular type of allergy sensitization. The y-axis shows the percentages of children with positive allergic sensitization in either group.

**Figure 3:**
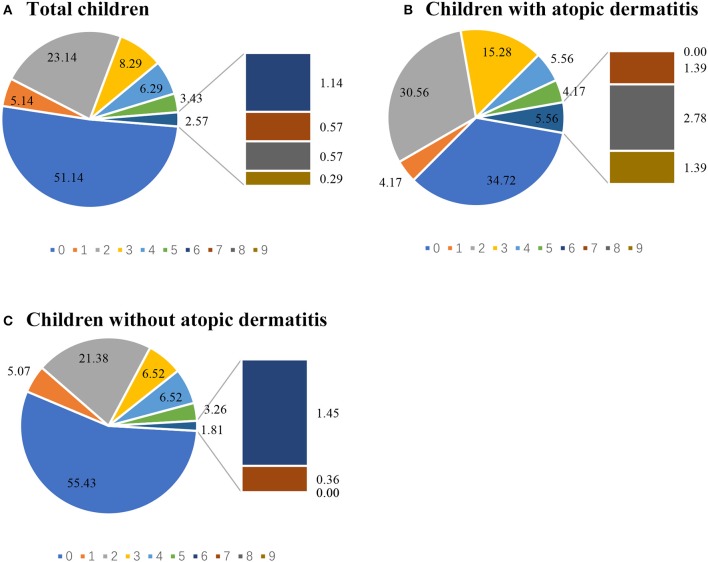
The positive number of allergic sensitization in each child was calculated. Percentages of children with particular number of allergic sensitization were analyzed in all children with skin prick test **(A)**, children with AD and underwent skin prick test **(B)**, and children without AD and underwent skin prick test **(C)** which was calculated as percentage(%)=(number of children with particular number of allergic sensitization)/(the number of children in each group who underwent the skin prick test) × 100%.

A total of 444 (82.53%) children underwent skin prick tests. Inhaled allergens were the most common allergen. More than 40% of children were sensitive either to *Der p* (195/444, 43.92%) or to *Der f* (200/444, 45.05%), and about 10% were sensitive to dog dander (42/444, 9.46%) or cat dander (40/444, 9.01%), respectively (Table 2). Among AD cases, the proportions of children with positive sensitization to *Der p* as well as *Der f* were about 1.5 times higher than those of the non-AD group. The percentage of children with positive sensitization to dog and cat dander was comparable in AD and non-AD group (Figure 2). Percentages of children with a particular number of allergic sensitizations are shown in Figure 3. The percentage of SPT positive reactors at 5 years was 65.28% in the group of children with AD, higher than that in the non-AD group (44.57%). The proportion of children who developed sensitization to two or more allergens was 61.11% in AD group, which was also much higher than that in the non-AD group (39.50%). Percentage of SPT negative reactors was lower in AD group than that in non-AD group (34.72 vs. 55.43%).

**Table 2:** Prevalence of positive allergic sensitization in all children, atopic dermatitis group, and non-atopic dermatitis group.

**Allergens**	**Total (%)**	**AD-group (%)**	**Non AD-group (%)**
Dermatophagoides pteronyssinus	43.92	60.61	39.13
Dermatophagoides farina	45.05	61.62	40.29
Dog	9.46	11.11	8.99
Cat	9.01	8.08	9.28
Aspergillus niger	2.93	6.06	2.03
Ragweed pollen	1.35	1.01	1.45
Platanus hispanica pollen	1.35	0.00	1.74
Birch pollen	2.03	3.03	1.74
Willow pollen	3.38	7.07	2.32
Egg	3.38	8.08	2.03
Milk	2.25	4.04	1.74
Mango	0.90	3.03	0.29
Prawn	2.25	8.08	0.58
Sea crab	1.80	6.06	0.58
Beef	1.13	1.01	1.16
Mutton	0.90	1.01	0.87
Cashew	0.90	3.03	0.29
Walnut	2.48	4.04	2.03

Pet or dog ownership during the infancy period was associated with increased sensitization to dog (OR _adjusted_ = 2.85, [95%CI: 1.08–7.50] for pet ownership and OR _adjusted_ = 3.44, [95%CI: 1.31–9.07] for dog ownership) (Table 3). The similar positive associations were observed between pet and dog ownership during the preschool period and the incidence of dog allergies (OR _adjusted_ = 2.73, [95%CI: 1.33–5.61] for pet ownership and OR _adjusted_ = 3.64, [95%CI: 1.57–8.42] for dog ownership). Prenatal pet or dog ownership was also related to increased prevalence of dog allergies. However, the association did not reach statistical significance. Similar situation was found in the association between pet or dog ownership and the prevalence of cat allergies (Table 3).

**Table 3:** Associations between early life pet ownership and the risk of sensitization to dog and cat at 5 years of age.

	**Sensitization outcome at age of 5 years**					
	**Dog sensitization**			**Cat sensitization**		
	**N(%)**	**Crude ORs (95%CIs)**	**Adjusted ORs (95%CIs)**	**N(%)**	**Crude ORs (95%CIs)**	**Adjusted ORs (95%CIs)**
**PET OWNERSHIP**						
During pregnancy	6 (11.54)	1.31 (0.52–3.29)	1.48 (0.56–3.90)	6 (11.54)	1.36 (0.54–3.39)	1.36 (0.52–3.55)
Infant period	8 (16.00)	**2.50 (1.03**–**6.03)**	**2.85 (1.08**–**7.50)**	8 (16.00)	**2.50 (1.03**–**6.03)**	2.15 (0.82–5.46)
Preschool period	14 (18.18)	**2.67 (1.33**–**5.35)**	**2.73 (1.33**–**5.61)**	9 (11.69)	1.42 (0.65–3.12)	1.43 (0.64–3.17)
**DOG OWNERSHIP**						
During pregnancy	6 (13.04)	1.50 (0.59–3.77)	1.73 (0.65–4.64)	6 (13.04)	1.59 (0.63–4.03)	1.58 (0.60–4.17)
Infant period	8 (18.18)	**2.98 (1.22**–**7.26)**	**3.44 (1.31**–**9.07)**	8 (18.18)	**2.98 (1.22**–**7.26)**	**2.72 (1.07**–**6.90)**
Preschool period	10 (22.22)	**3.25 (1.48**–**7.16)**	**3.64 (1.57**–**8.42)**	6 (13.33)	1.64 (0.65–4.15)	1.62 (0.63–4.17)

*Adjusted OR: calculated by the logistic models adjusted for sex, maternal atopy, maternal education, mode of delivery, birthweight, parity, gestational age, and smoking during pregnancy*.

*Bold values indicate with statistical significance*.

### Associations Between Early Life Pet Ownership and AD at 5 Years

Of 538 children, 112 (20.82%) were diagnosed with AD at 5 years of age. As illustrated in Table 4, pet ownership during infancy was significantly associated with a lower risk of AD at 5 years (OR _adjusted_ = 0.33, 95% CI: 0.12–0.88). The risk of AD was also halved in the group of preschool children exposed to pet during pregnancy and during the preschool period. However, the association did not reach statistical significance possibly due to the limited exposure. These associations persisted after adjustment for covariates as mentioned in methods. When analyzing association between dog ownership and risk of AD, the trend was similar to that between pet ownership and risk of AD. We also evaluated whether pet ownership could affect the severity of AD. The average SCORAD index of patients with pet exposure during infancy was 18.49 (range: 0.02–35.10) and without pet exposure was 17.15 (range: 6.27–40.40). No difference was found in the SCORAD index between children with pet exposure during infancy and those without (*p* = 0.39, *p* > 0.05), neither between children with and without dog exposure (19.40, 9.90–35.10 vs. 16.99, 0.02–40.40) (*p* = 0.15, *p* > 0.05). Similar non-significant results were also found between AD children with pet exposure and those without exposure at pregnancy and in preschool period.

**Table 4:** Associations between early life pet ownership and the risk of atopic dermatitis at 5 years of age.

	**N(%)**	**Crude ORs**	**95% CI**	**Adjusted ORs**	**95% CI**
**Pet ownership**					
During pregnancy	8 (13.33)	0.47	0.21–1.05	0.51	0.22–1.18
Infant period	6 (10.91)	**0.34**	**0.13–0.88**	**0.33**	**0.12–0.88**
Preschool period	14 (15.22)	0.64	0.35–1.17	0.62	0.33–1.16
**Dog Ownership**					
During pregnancy	7 (13.21)	0.55	0.24–1.26	0.60	0.25–1.40
Infant period	5 (10.64)	**0.32**	**0.11–0.92**	**0.31**	**0.11–0.91**
Preschool period	9 (17.65)	0.8	0.38–1.69	0.72	0.33–1.57

*Pet ownership was defined as the presence of any furred pet living in the house at the time of visit*.

*Adjusted OR: logistic models adjusted for sex, maternal atopy, maternal education, mode of delivery, birthweight, parity, gestational age, and passive smoking during pregnancy*.

*Bold values indicate with statistical significance*.

We further analyzed the association between dog sensitization and AD according to dog ownership (Table 5). Among children with dog ownership, the percentage of AD was much lower in the group with dog sensitization than those without (Table 5). Dog allergies decreased the risk of AD in children who raised dog at home (OR = 0.82, [95%CI: 0.71–0.94] for pregnancy exposure; the OR was 0.89, [95%CI: 0.79–1.00] for infancy exposure and 0.78 [95%CI: 0.66–0.93] for preschool exposure), but the dog sensitization had no association with the risk of AD in children without dog ownership (Table 5). However, no significant difference was found between cat sensitization and AD according to cat ownership (data not shown).

**Table 5:** Association between dog sensitization and risk of AD at age of 5 years according to the dog ownership status (YES/NO) in early life.

	**Dog sensitization at 5 years of age**		**OR (95% CI)**
	**Yes (Cases/N)**	**No (Cases/N)**	
**Dog ownership**			
During pregnancy	0/6	6/41	**0.82 (0.71–0.94)**
Infant period	0/8	5/36	**0.89 (0.79–1.00)**
Preschool period	2/10	6/35	**0.78 (0.66–0.93)**
**No dog ownership**			
During pregnancy	11/36	82/361	0.66 (0.32–1.41)
Infant period	8/20	59/269	0.43 (0.17–1.11)
Preschool period	9/32	82/367	0.74 (0.33–1.65)

*Bold values indicate with statistical significance*.

## Discussion

This is the first prospective birth cohort study to investigate the relationship between pet ownership and allergic sensitization and AD in Chinese population. We found that more than 40% of children had positive allergic sensitization in Shanghai, and almost one fifth of them suffered from AD. Children with infancy and preschool pet and dog ownership tended to have the dog sensitization. However, pet ownership during infancy was associated with a lower risk of developing AD at age 5 years.

To date, no birth cohort study in Shanghai had reported epidemiological data of allergic sensitization. Dermatophagoides pteronyssinus or Dermatophagoides farina were the most common allergens in our study, similar to previously reported data in Asian countries. (22) Dog and cat dander sensitization ranked the second. In a previous cross-sectional study in Shanghai, mite allergy was also the most common allergen (17.6%), followed by pollen (12.8%), and food allergy (12.3%) (23). Prevalence of overall European rates of sensitization to cat allergens were 26.3%, ranging from 16.8 to 49.3% and to dog allergens 27.2%, ranging from 16.1 to 56.0%, much higher than that to Dermatophagoides pteronyssinus and Dermatophagoides farina (1–7%). (24) Overall, the percentage of SPT positive reactors in the group of children with AD was higher than that in non-AD group. Cumulative evidence revealed that dog exposure in early childhood prevents the development of allergies to dogs and cats (5, 12, 13). However, a positive association between postnatal pet exposure and the prevalence of dog sensitization was found in our study, which was consistent with previous data (16, 17, 25, 26).

It is reported that pet-or dog-ownership during pregnancy significantly lowered the risk of AD in the year of 1 or 2 in two previous prospective birth cohort studies (10, 27). Roduit et al. found that children with prenatal pet and cat contact had a lower risk of AD in the first 2 years of life than those without (10). Another study regarding prenatal maternal exposure to dogs at home was “protective” against allergic diseases (27). Similarly, the risk of AD was also lower in the group of pet ownership during pregnancy in this study although it did not reach statistical significance. It is possibly due to the small number of children with prenatal pet and dog exposure.

Inconsistent results were found in correlation between postnatal pet exposure and development of allergic diseases in previous studies (5–7, 12, 13, 28–31). The probable causes for these discrepancies could be the differences in the timing of exposures, the way pets are kept and the diagnosis criteria of AD (11). Pet exposure was not well-measured as pet contact was considered as pet exposure in some studies, which was very difficult to quantify. The exposure window span was too wide from pregnancy to infancy and even the toddler period. In addition, the diagnosis of AD in previous literature was mostly measured by questionnaire (32). It's hard to make diagnosis correctly without disease history and physical examination. In this birth cohort study, with thorough skin physical examination and lab tests like skin prick test, we diagnosed AD and found a significant association between pet ownership during the infancy period and the prevalence of AD.

Early life represents a critical window for immune development. During this period, a variety of environmental factors, such as pet exposure, encountered by infants determine health or allergic diseases (33). Several protective mechanisms of early life pet exposure had been summarized as follows. Exposure to endotoxin has been speculated as the cause behind the protective effect, which may shift the developing immune system to predominantly Th1-type responses that protect children from developing allergy (34–37). The study by Gern et al. observed the association between dog ownership and higher serum IL-10 and IL-13 cytokine secretion in children at age 1 (5). Cytokine balance during early life, which could be influenced by external factors, is critical in the development of allergic response (30). In addition, recent studies attempting to correlate genetic variations in Toll-like receptors (TLRs) and CD14 genes with atopic disease supported the existence of a gene-environment interaction, although the results were conflicting (5, 10, 33). However, research by Swedish BAMSE presented that the observed protective effect of dog exposure may be partly due to selective dog avoidance by families with parental atopic dermatitis (38).

In our study, pet ownership during infancy was associated with a lower risk of children AD at age 5 years, but with a higher risk of dog sensitization. Constant exposure to high levels of animal antigen over time might induce tolerance to relevant allergen in sensitized children via desensitization of mast cells and basophils, generation of allergen-specific Treg and Breg cells, inhibition of Th2 cells and other allergen-specific effector T cells. It also could be via the regulation of antibody isotypes with increase in specific IgG4 and decrease or no change in specific IgE (39). Dog sensitization may persist with constant exposure to animals, but children will not develop AD. The relationship between pet exposure, dog sensitization and the prevalence of AD in our study may be explained by the above-mentioned mechanisms.

There are some limitations in this study. As it usually occurs in other longitudinal cohort studies, some participants were lost during the follow-up, resulting in a relatively small sample size in this cohort. The follow up rate was only 70.56% in our study. The number of families with cat/dog exposure was also small in this study, which may cause deviations to the results. In addition, although we adjusted for a wide range of potential confounders including maternal atopy, possible existence of previous family history of allergic disease can cause bias. Another limitation is that this study failed to explain the observed effect of pet ownership on sensitization and some subjects did not undergo the SPT test. Finally, there may be some residual confounding factors that the data do not allow us to adjust, although several potential confounders had been analyzed. All data were collected prospectively, and hence, recall bias of the information did not exist.

In conclusion, the present cohort study found that pet ownership in early childhood might potentially protect children from the development of AD up to the age of 5 years. Postnatal exposure to a pet at home increased the risk of sensitization to dogs. Further well-designed, large scaled, prospective investigations on the protective effect of pet ownership are needed.

## Data Availability Statement

The datasets generated and/or analyzed during the current study are not publicly available due to management rules by the study funder but are available from the corresponding author on reasonable request.

## Ethics Statement

The studies involving human participants were reviewed and approved by the Bioethical Committee of the institutional review board of Xinhua hospital and the International Peace Maternity and Child Hospital, shanghai, China. Written informed consent to participate in this study was provided by the participants' legal guardian/next of kin.

## Author Contributions

CL: conceptualization, methodology, original draft preparation, and funding acquisition. QC: conceptualization, methodology, software, formal analysis, and data curation. XZ, HL, QL, and PF: investigation, validation, writing—review, and editing. LH and ZY: funding acquisition, supervision, and project administration.

## Conflict of Interest

The authors declare that the research was conducted in the absence of any commercial or financial relationships that could be construed as a potential conflict of interest.
